# Differential role of MLKL in alcohol-associated and non–alcohol-associated fatty liver diseases in mice and humans

**DOI:** 10.1172/jci.insight.140180

**Published:** 2021-02-22

**Authors:** Tatsunori Miyata, Xiaoqin Wu, Xiude Fan, Emily Huang, Carlos Sanz-Garcia, Christina K. Cajigas-Du Ross, Sanjoy Roychowdhury, Annette Bellar, Megan R. McMullen, Jaividhya Dasarathy, Daniela S. Allende, Joan Caballeria, Pau Sancho-Bru, Craig J. McClain, Mack Mitchell, Arthur J. McCullough, Svetlana Radaeva, Bruce Barton, Gyongyi Szabo, Srinivasan Dasarathy, Laura E. Nagy

**Affiliations:** 1 Northern Ohio Alcohol Center, Department of Inflammation and Immunity, Cleveland Clinic, Cleveland, Ohio, USA.; 2 Department of Gastroenterological Surgery, Kumamoto University Hospital, Kumamoto, Japan.; 3 Department of Molecular Medicine and; 4 Department of Family Medicine, Metro Health Medical Center, Case Western Reserve University, Cleveland, Ohio, USA.; 5 Department of Pathology, Cleveland Clinic, Cleveland, Ohio, USA.; 6 Institut d’Investigacions Biomèdiques August Pi i Sunyer (IDIBAPS), Hospital Clinic of Barcelona, Barcelona, Spain.; 7 Centro de Investigación Biomédica en Red de Enfermedades Hepáticas y Digestivas (CIBERehd), Barcelona, Spain.; 8 Department of Medicine, University of Louisville, Louisville, Kentucky, USA.; 9 Internal Medicine, University of Texas Southwestern Medical Center, Dallas, Texas, USA.; 10 Department of Gastroenterology and Hepatology, Cleveland Clinic, Cleveland, Ohio, USA.; 11 National Institute on Alcohol Abuse and Alcoholism, Bethesda, Maryland, USA.; 12 Department of Population and Quantitative Health Sciences, University of Massachusetts Medical School, Worcester, Massachusetts, USA.; 13 Department of Medicine, Beth Israel Deaconess Medical Center and Harvard Medical School, Boston, Massachusetts, USA.; 14 Department of Gastroenterology and Hepatology, Cleveland Clinic, Cleveland, Ohio, USA.

**Keywords:** Hepatology, Inflammation, Hepatitis

## Abstract

Hepatocellular death contributes to progression of alcohol–associated (ALD-associated) and non–alcohol-associated (NAFL/NASH) liver diseases. However, receptor-interaction protein kinase 3 (RIP3), an intermediate in necroptotic cell death, contributes to injury in murine models of ALD but not NAFL/NASH. We show here that a differential role for mixed-lineage kinase domain–like protein (MLKL), the downstream effector of RIP3, in murine models of ALD versus NAFL/NASH and that RIP1-RIP3-MLKL can be used as biomarkers to distinguish alcohol-associated hepatitis (AH) from NASH. Phospho-MLKL was higher in livers of patients with NASH compared with AH or healthy controls (HCs). MLKL expression, phosphorylation, oligomerization, and translocation to plasma membrane were induced in WT mice fed diets high in fat, fructose, and cholesterol but not in response to Gao-binge (acute on chronic) ethanol exposure. *Mlkl*^–/–^ mice were not protected from ethanol-induced hepatocellular injury, which was associated with increased expression of chemokines and neutrophil recruitment. Circulating concentrations of RIP1 and RIP3, but not MLKL, distinguished patients with AH from HCs or patients with NASH. Taken together, these data indicate that MLKL is differentially activated in ALD/AH compared with NAFL/NASH in both murine models and patients. Furthermore, plasma RIP1 and RIP3 may be promising biomarkers for distinguishing AH and NASH.

## Graphical abstract

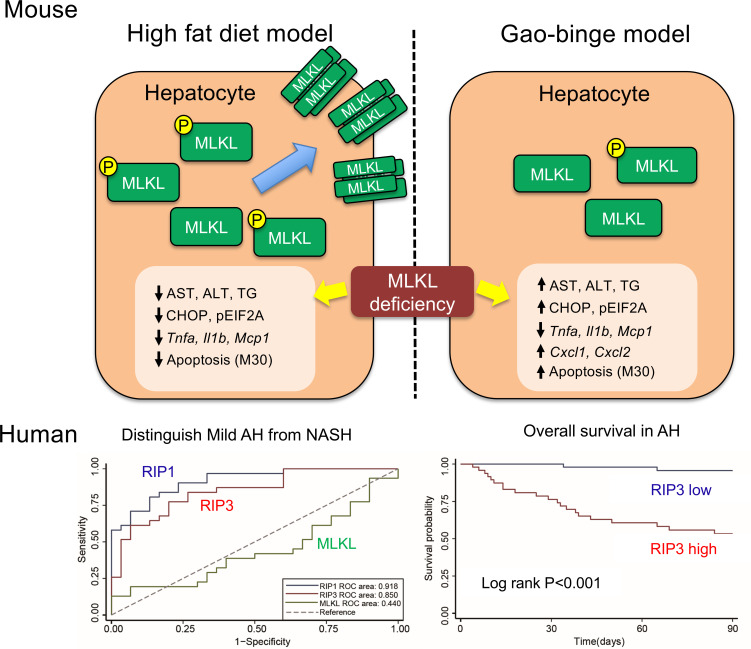

## Introduction

Alcohol-associated liver disease (ALD) and non–alcohol-associated fatty liver/steatohepatitis (NAFL/NASH) are major causes of liver disease in the world (1). Indeed, mortality from ALD and NAFL/NASH is increasing compared with that resulting from viral hepatitis, owing to effective therapeutics and lifestyle changes (1). ALD and NAFL/NASH have similar pathological spectra, ranging from simple steatosis to hepatitis to cirrhosis and increased risk for hepatocellular carcinoma (1, 2). Progression of liver disease involves complex crosstalk between parenchymal and nonparenchymal cells resident in the liver, as well as the recruitment of immune cells to the liver in response to injury.

The intricate balance between cell death and prosurvival pathways is critical for regulating inflammation and hepatocellular injury during progression of ALD or NAFL/NASH. Four major forms of regulated cell death are active in hepatocytes, including apoptosis, necroptosis, ferroptosis, and pyroptosis (3). Recent studies have focused on the role of necroptosis in murine models of ALD and NAFL/NASH. Necroptosis is a proinflammatory mode of programmed cell death that is an alternative to apoptotic cell death. Necroptosis is morphologically similar to necrosis, with cell swelling and rupture, releasing potentially proinflammatory cellular contents. In contrast, apoptotic cells condense and cellular contents remain trapped in vesicular structures. Although apoptosis requires the activation of caspases, necroptosis is driven by the activity of receptor-interaction protein kinase 1 and 3 (RIP1 and RIP3), resulting in the phosphorylation, oligomerization, and translocation of mixed-lineage kinase domain–like protein (MLKL) to the plasma membrane (PM) (4–7). Necroptosis is activated by death receptor activation and innate immune signals, including TNF-α, TLR3, and TLR4 ligands, as well as DNA-dependent activator of IFN regulatory factors (DAI) (4–7).

Studies using *Rip3*^–/–^ mice, RIP1 kinase dead mice, and RIP1 kinase inhibitors identified differential contributions of RIP1 and RIP3 to the progression of liver injury in multiple murine models of liver diseases (4–10). *Rip3*^–/–^ mice are protected from chronic ethanol-induced liver injury (8, 11), as well as acetaminophen-induced hepatotoxicity (12, 13), methionine-choline deficient (MCD) diet-induced NAFL/NASH (14), and concanavalin A-induced autoimmune hepatitis (15). However, work by our lab (16) and Gautheron and colleagues (17, 18) found *Rip3*^–/–^ mice were not protected from high-fat diet–induced liver injury. The differential contribution of RIP3 is of considerable interest, as it highlights specific pathophysiological mechanisms for liver diseases of different etiologies (19).

Although the contribution of *Rip3* to different liver diseases has been studied, the role of *Mlkl* in liver diseases has not been broadly investigated. Additionally, MLKL was found to be involved in the development of obesity-induced insulin sensitivity in liver, but had only a minor effect on hepatic inflammation (20). Recently, we reported that MLKL-deficient (*Mlkl*^–/–^ ) mice were protected from liver injury in a diet high in fat, fructose, and cholesterol–induced (FFC-induced) (typical Western diet) model of obesity (21). Importantly, MLKL was activated and oligomerized at the plasma membrane in liver independently of *Rip3* in response to FFC diets (21), consistent with the lack of protection from high-fat diets observed in *Rip3*^–/–^ mice (16–18). In addition, this *Rip3*^–/–^-independent role of MLKL in FFC diet-induced liver injury was associated with impaired autophagic flux (21).

Based on the available studies delineating the differential contributions of RIP1-/RIP3-MLKL signaling pathway in murine models of ALD and NAFL/NASH, here we investigated the role of *Mlkl* in ethanol-induced liver injury compared with its role in response to high-fat diet–induced liver injury. In contrast to the protection of *Mlkl*^–/–^ from liver injury in response to FFC diet (21); *Mlkl-*deficient mice were not protected from either chronic ethanol or acute on chronic (Gao-binge) ethanol-induced liver injury. Although the Gao-binge–induced expression of inflammatory cytokines in the liver was decreased in *Mlkl*^–/–^ mice, *Mlkl*-deficiency did not protect from *Cxcl* family chemokine expression and neutrophil accumulation, steatosis, ER stress, or hepatocellular injury. Importantly, immunoreactive phospho-MLKL (pMLKL) was higher in livers of patients with NASH compared with patients with AH or healthy controls (HCs), and circulating concentrations of RIP1 and RIP3 distinguished patients with AH from NASH or HCs. Taken together, these data highlight that the molecular machinery in the RIP3-MLKL signaling network differentially contribute to murine models of AH and NAFL/NASH and that RIP1 and RIP3 might be useful biomarkers to distinguish AH from NASH.

## Results

### Differential expression and phosphorylation MLKL in livers of patients with AH and NASH compared with HCs.

**Figure 1:**
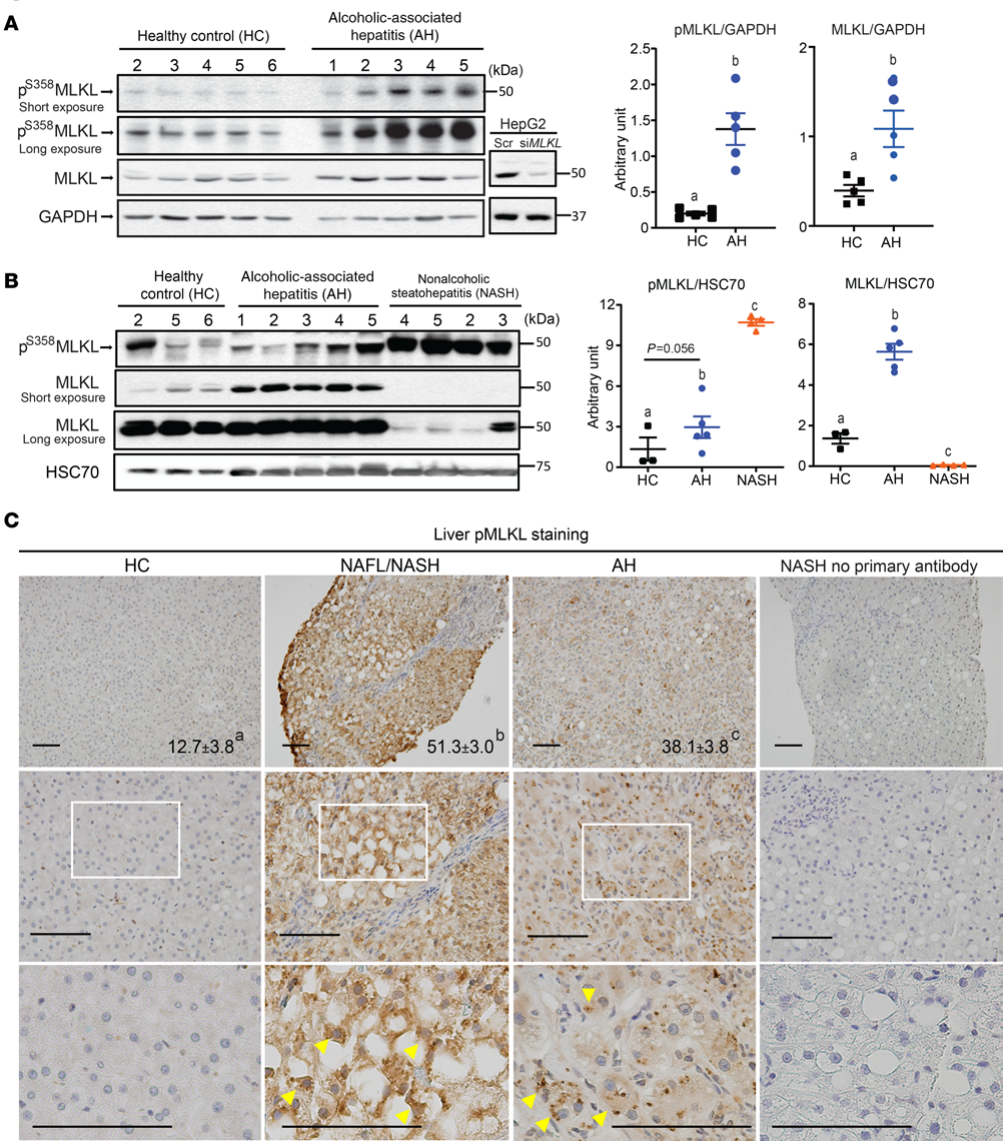
MLKL and pMLKL expression in liver in patients with alcohol-associated hepatitis and nonalcoholic steatohepatitis. (**A** and **B**) Western blot analysis of MLKL and pMLKL expression in liver lysates from healthy controls (HCs) and patients with alcohol-associated hepatitis (AH) (*n* = 5) (**A**) or HCs and patients with nonalcoholic steatohepatitis (NASH) and AH (*n* = 4–5) in liver lysates (**B**). HepG2 transfected with siRNA targeting *MLKL* were used as negative controls. (**C**) Paraffin-embedded human liver samples were deparaffinized followed by pMLKL immunostaining using standard immunohistochemistry technique. Nuclei were counterstained with hematoxylin. For a negative control, a liver sample from a patient with NASH was processed without primary antibody. Arrowheads show typical pMLKL-positive area. pMLKL-positive areas were quantified using Image Pro-Plus software, excluding any tissues edges from the quantification. Images are representative on HC (*n* = 10), NAFL/NASH (*n* = 16), and AH (*n* = 10). Scale bar: 100 μm. *P* less than 0.05, assessed by 2-way ANOVA; values with different alphabetical superscripts were significantly different from each other.

RIP3 expression is low in healthy livers (4); however, expression is increased in livers of patients with ALD (8) and NAFL/NASH (14, 17). Although Afonso et al. (14) found somewhat elevated RIP3 in AH patients compared with NASH via immunohistochemistry, no data are available on direct comparisons of MLKL expression between patients with different etiologies of liver disease. We therefore evaluated the phosphorylation of MLKL in livers from patients with AH and NASH compared with HCs by Western blot (Figure 1, A and B) and immunohistochemistry (Figure 1C). Clinical and demographic data are provided in Table 1.

Liver homogenates from patients with AH and HCs were semiquantified on the same Western blot. The relative quantity of MLKL and pMLKL were higher in AH compared with controls (Figure 1A). Next, a subset of these HCs and patients with AH were directly compared on a single Western blot to patients with NASH (Figure 1B). As in Figure 1A, pMLKL was higher in patients with AH compared with HCs, pMLKL was higher in NASH compared with both AH and HCs (Figure 1B). In contrast, the relative total MLKL expression was lower in NASH compared with AH and HCs. HepG2 cells transfected with siRNA targeted to *MLKL* were used as negative controls for detection of MLKL (Figure 1A). Consistent with the Western blot data, pMLKL, assessed by immunohistochemistry, was higher in NASH and AH compared with HCs and also higher in NASH compared with AH (Figure 1C). Interestingly, the distribution of pMLKL across the parenchyma was also different between AH and NASH. In NASH, pMLKL-positive cells showed relatively uniform staining in the cytoplasm, whereas in AH, pMLKL-positive cells exhibited a more punctate staining pattern (Figure 1C). Taken together, these data suggest differential regulation of MLKL in livers from patients with AH and NASH.

### Differential expression and distribution of MLKL in liver of ethanol-fed versus FFC diet-fed mice.

**Figure 2:**
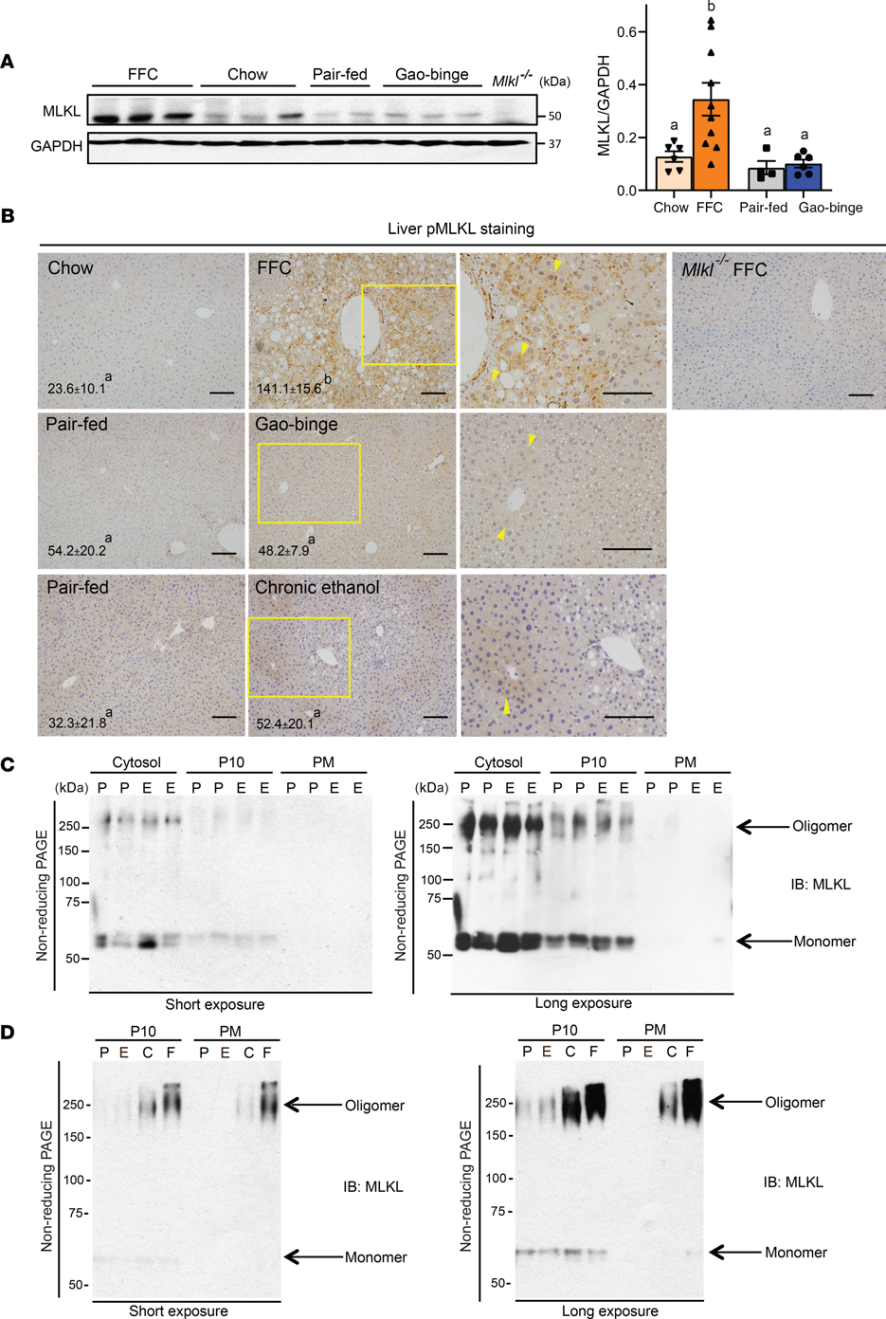
Differential expression and activation of hepatic MLKL in mice exposed to Gao-binge ethanol diet compared with FFC diet. For a model of NAFL/NASH *Mlkl^+/+^* mice were allowed free access to a Western diet high in fat, fructose, and cholesterol (FFC) for 12 weeks or standard chow diets. For a model of ALD, mice were allowed free access to the Lieber-DeCarli ethanol diet or pair-fed control diet for 10 days followed by an acute gavage of ethanol or maltose, respectively. (**A**) Western blot analysis of MLKL was measured in livers from mice fed the FFC diet or Gao-binge ethanol model. A liver sample from an *Mlkl*^–*/*–^ mouse fed the FFC diet was used as a negative control. *n* = 4–10 per group. (**B**) Paraffin-embedded liver samples were deparaffinized and stained for pMLKL. Nuclei were counterstained with hematoxylin. Arrowheads show phospho-MLKL (pMLKL)-positive areas. An *Mlkl*^–*/*–^ mouse exposed to the high-fat diet feeding protocol was used as a negative control. Representative images are shown. *n* = 4–11 per group. Scale bar: 100 μm. *P* less than 0.05, assessed 2-tailed *t* test (**A**) and 2-way ANOVA (**B**); values with different alphabetical superscripts were significantly different from each other. (**C** and **D**) Oligomerization and subcellular localization of MLKL in the livers of mice were assessed in the cytosol, 10,000*g* pellets (P10) and isolated plasma membranes (PMs). Proteins were resolved by nonreducing PAGE and probed with antibody to MLKL. A longer exposure is shown in the right panel to better illustrate MLKL in the PM fraction. (**C**) Distribution of MLKL monomer and oligomer in liver of mice exposed to Gao-binge ethanol. (**D**) Distribution of MLKL monomer and oligomer in liver of mice exposed to Gao-binge ethanol compared with FFC diet. Representative images are shown. C, chow; E, Gao-binge; F, FFC; P, pair-fed.

If MLKL, like RIP3, plays a differential role in ethanol-induced compared with FFC diet-induced liver injury in mice, then activation of MLKL would be expected to differ between these 2 disease models. To test this hypothesis, MLKL expression in liver was compared between mice challenged with the Gao-binge model of ethanol exposure and mice fed a FFC diet (Figure 2A). FFC diet, but not the Gao-binge ethanol protocol, increased immunoreactive MLKL expression (Figure 2A). Activation of MLKL involves phosphorylation, oligomerization, and translocation to the PM (22). pMLKL expression, assessed by immunohistochemistry, was also increased in response to the FFC diet, but not Gao-binge or chronic ethanol exposure (Figure 2B). We recently reported that feeding FFC diet induced the oligomerization and translocation of MLKL to the plasma membrane fraction in mouse liver (21). In contrast, here we found that MLKL did not oligomerize or translocate to the PM in response to Gao-binge ethanol exposure (Figure 2C). The lack of translocation was particularly evident when compared directly to the impact of FFC diet to increase MLKL oligomers in the PM fraction (Figure 2D).

### Mlkl deficiency does not protect mice from indicators of Gao-binge–ethanol- or chronic ethanol-induced liver injury.

**Figure 3:**
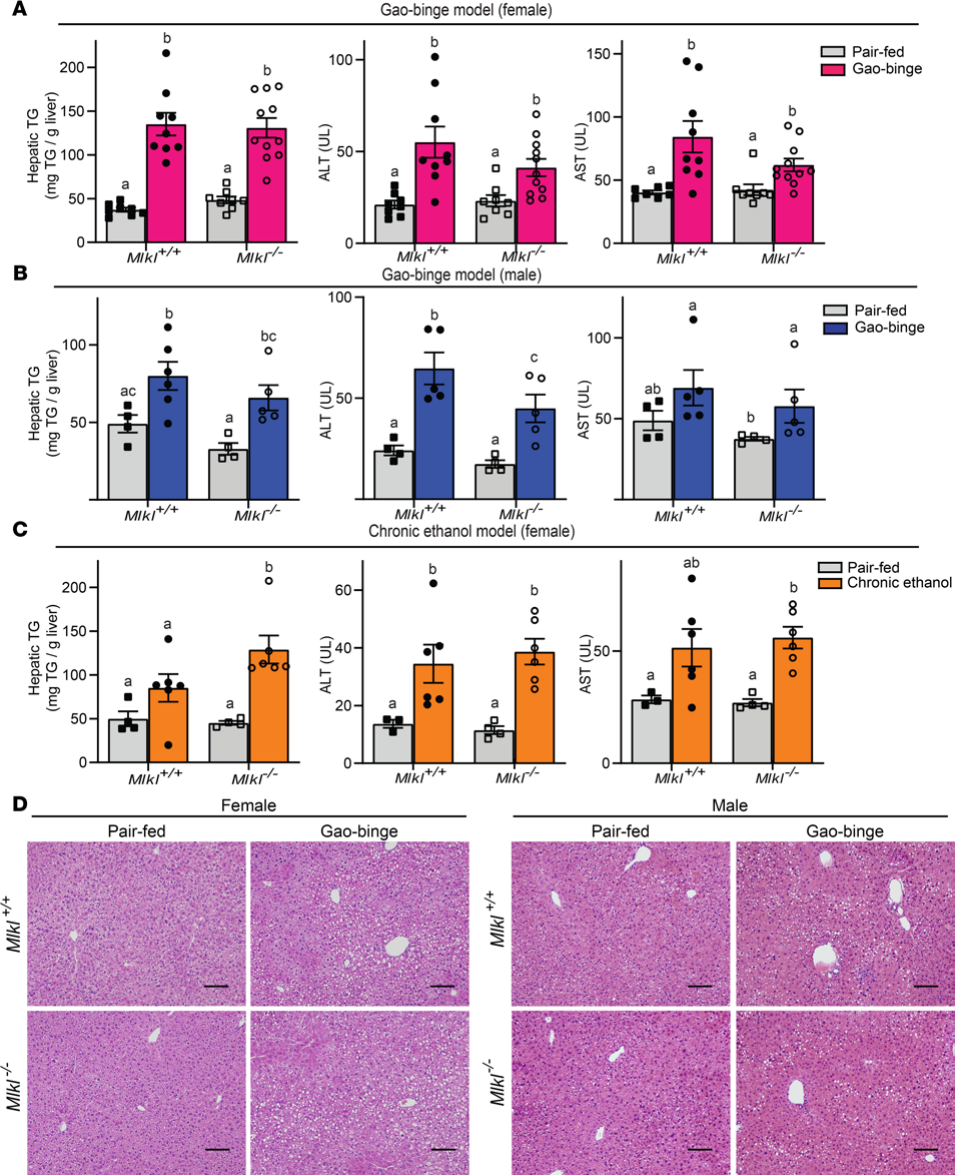
*Mlkl*^–/–^ mice are not protected from Gao-binge– or chronic ethanol-induced liver injury. (**A**–**C**) Female (**A**) and male (**B**) MLKL-deficient mice (*Mlkl*^–/–^) and their littermate controls (*Mlkl^+/+^*) were exposed to the Gao-binge ethanol diet as described in Figure 2 and chronic ethanol (**C**) as described in Supplemental Material. ALT and AST activities were measured in plasma, and triglyceride concentrations measured in liver. For females, *n* = 8–12 (**A**) and males, *n* = 4–6 (**B**) and female, *n* = 4–6 (**C**). *P* less than 0.05, assessed by 2-way ANOVA; values with different alphabetical superscripts were significantly different from each other. (**D**) H&E staining of livers in Gao-binge ethanol diet. Scale bar: 100 μm. Images are representative on *n* = 4–6 mice per group.

The low level of pMLKL in AH compared with NASH in both murine models and patients’ livers suggested that MLKL might not make a significant contribution to ethanol-induced liver injury. To evaluate the role of MLKL in murine models of ALD, *Mlkl*^–/–^ mice and their littermate controls (*Mlkl*^+/+^) were exposed to the Gao-binge or a chronic model of ethanol feeding. There were no major differences in body weights or food intake between genotypes (Supplemental Table 1; supplemental material available online with this article; https://doi.org/10.1172/jci.insight.140180DS1). Gao-binge ethanol exposure increased ALT, AST, and hepatic triglycerides in both female (Figure 3A) and male (Figure 3B) compared with pair-fed controls independently of *Mlkl* genotype. Similarly, chronic ethanol feeding increased hepatic triglycerides, ALT, and AST in both *Mlkl*^+/+^ and *Mlkl*^–/–^ mice (Figure 3C). Moreover, H&E staining of liver sections revealed a similar pattern of macrovesicular and microvesicular steatosis in males and females of both genotypes in response to Gao-binge ethanol (Figure 3D). Histological scoring of the H&E-stained liver sections was consistent with the biochemical assessment of liver injury (Supplemental Table 2).

### Gao-binge–induced ER stress and hepatocyte apoptosis were independent of Mlkl genotype.

**Figure 4:**
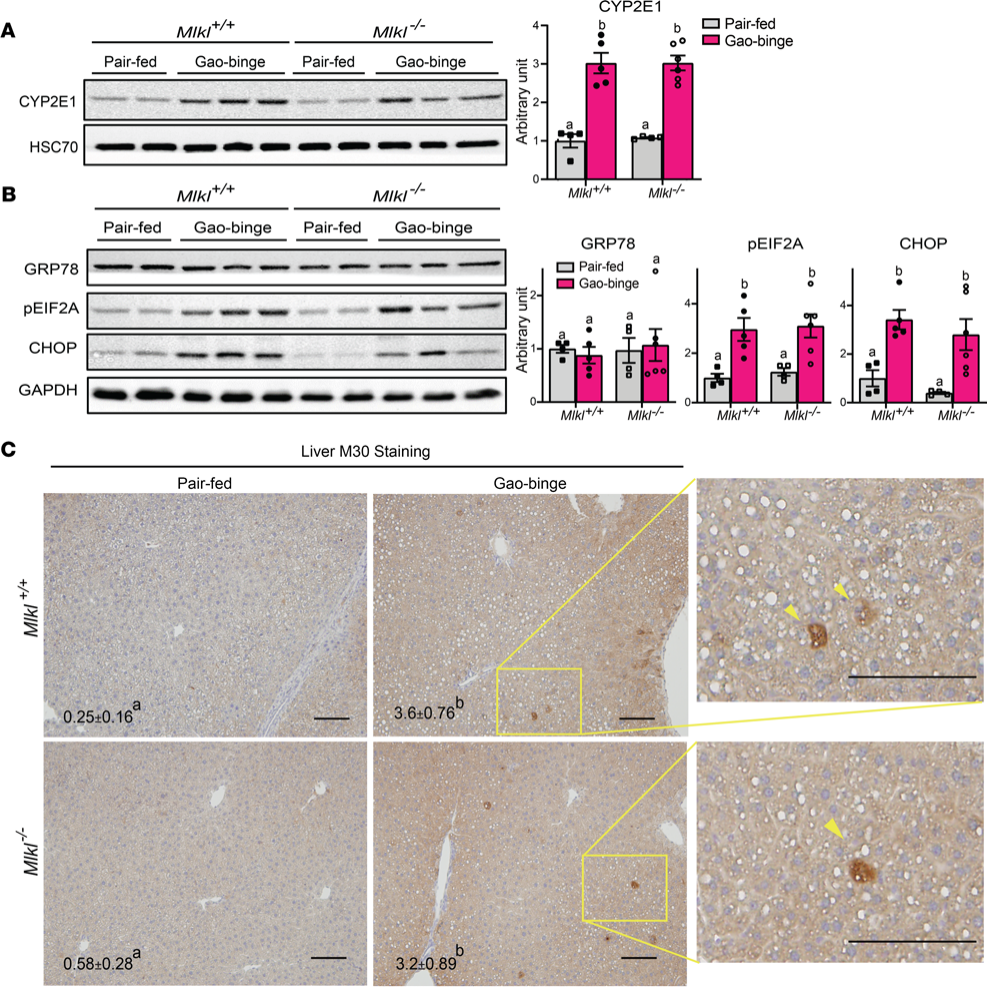
Differential role of *Mlkl* on Gao-binge–induced increases in ER stress and hepatocyte apoptosis. (**A** and **B**) Expression of CYP2E1 (**A**) and ER stress markers (**B**) were assessed by Western blot analysis and normalized to HSC70 or GAPDH. (**C**) M30-positive hepatocytes (total number of cells per 10X frame) were counted in formalin-fixed paraffin-embedded sections of liver. Arrowheads show typical M30-positive hepatocytes. Nuclei were counterstained with hematoxylin. Scale bar: 100 μm. *n* = 4–6 per group. *P* less than 0.05, assessed by 2-way ANOVA; values with different alphabetical superscripts were significantly different from each other.

Gao-binge–induced liver injury is also associated with increased CYP2E1 expression, ER stress, and hepatocyte apoptosis. CYP2E1 induction by Gao-binge was independent of *Mlkl* genotype (Figure 4A). Similarly, phosphorylation of EIF2A and induction of CHOP, 2 indicators of ER stress were increased in both *Mlkl*^–/–^ mice and *Mlkl*^+/+^ (Figure 4B). Finally, accumulation of M30, a specific marker of caspase-dependent hepatocyte apoptosis, was increased by Gao-binge independently of genotype (Figure 4C). Collectively, these data using *Mlkl*^–/–^ mice indicate that *Mlkl* does not make a significant contribution to ethanol-induced liver injury.

### Role of Mlkl in inflammatory cytokine and chemokine expression and neutrophil infiltration in response to Gao-binge ethanol exposure.

**Figure 5:**
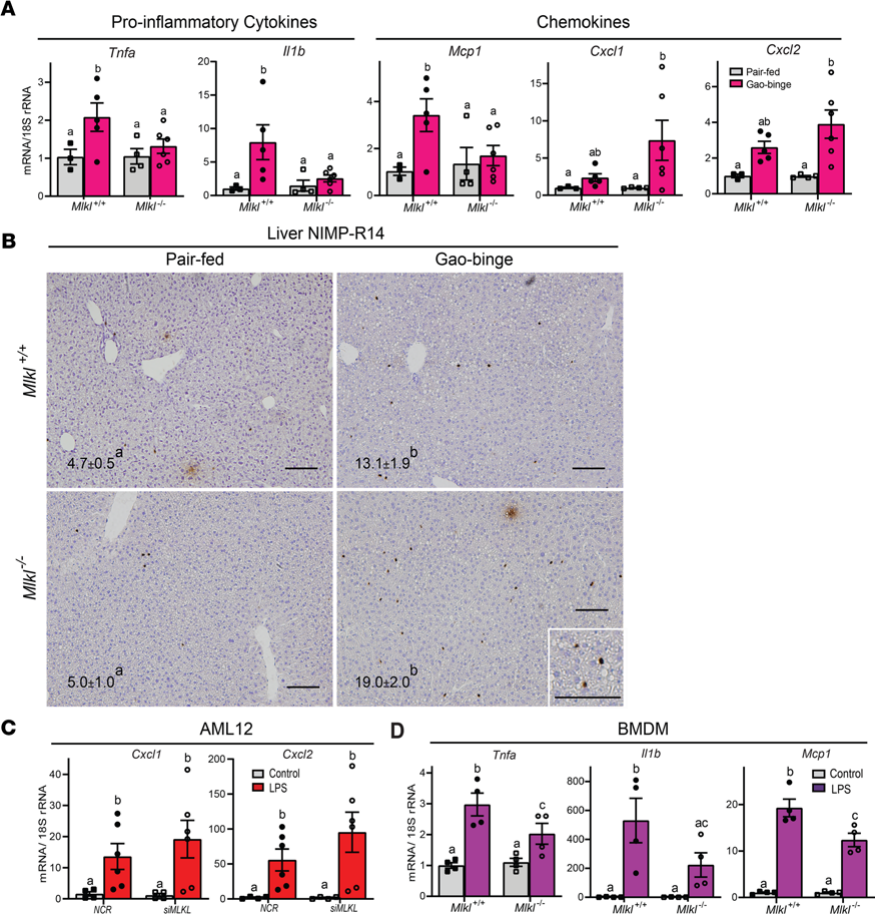
Differential role of Mlkl on Gao-binge–induced increases in inflammatory mediators and neutrophil accumulation. Female MLKL-deficient mice (*Mlkl*^–/–^) and their littermate controls (*Mlkl^+/+^*) were exposed to the Gao-binge ethanol diet as described in Figure 2. (**A**) mRNA expression for proinflammatory cytokines and chemokines was detected in livers from using quantitative real-time PCR (qRT-PCR) and normalized to 18S rRNA. (**B**) NIMP-R14-positive cells (total number of cells per 10X frame) were counted in formalin-fixed paraffin-embedded sections of liver. Nuclei were counterstained with hematoxylin. Scale bar: 100 μm. (**C**) Chemokine expression in AML12 transfected with scrambled siRNA (*NCR*) or siRNA targeting *Mlkl*. Cells were challenged with 10 ng/mL LPS for 1.5 hours and chemokine expression assessed by qRT-PCR. (**D**) Proinflammatory cytokine and chemokine expression in BM-derived macrophages (BMDMs) from *Mlkl*-deficient mice and WT controls. BMDMs were challenged with or without 10 ng/mL LPS for 24 hours and cytokine/chemokine expression assess by qRT-PCR. *n* = 4–6 per group for (**A**–**C**) and *n* = 4 for (**D**). *P* less than 0.05, assessed by 2-way ANOVA; values with different alphabetical superscripts were significantly different from each other.

Increased inflammatory cytokine expression has long been appreciated as an important contributor to ethanol-induced liver injury, owing, at least in part, to increased concentrations of LPS in the portal circulation, resulting from impaired gut barrier function in response to ethanol (23). More recently, it has become evident that dysregulated chemokine expression is also a key feature of ethanol-induced liver injury, particularly related to the infiltration of neutrophils (24–26). Gao-binge ethanol feeding increased *Tnfa, Il1b* and *Mcp1* expression in liver of *Mlkl^+/+^*, but not *Mlkl*^–/–^, mice (Figure 5A). In contrast, expression of the chemokines *Cxcl1* and *Cxcl2* was increased in response to Gao-binge independent of genotype (Figure 5A). Consistent with the sustained increase in *Cxcl1*/*Cxcl2*, neutrophil accumulation in response to Gao-binge ethanol was also independent of *Mlkl* genotype (Figure 5B).

Although immune cells in the liver are typically considered the primary source of inflammatory cytokines and chemokines, there is growing evidence that hepatocytes themselves are key sources of chemokines (27, 28). By isolating primary hepatocytes from nonparenchymal cells of livers of control mice, MLKL was expressed in both cell populations (Supplemental Figure 1). Therefore, we hypothesized that *Mlkl* might differentially contribute to cytokine and chemokine expression in hepatocytes compared with immune cells. Exposure of AML12 hepatocytes to low concentrations of LPS, similar to those observed in patients with AH or mice exposed to chronic ethanol, increased the expression of *Cxcl1* and *Cxcl2* expression. This response was maintained even when *Mlkl* was knocked down using targeted siRNAs (Figure 5C). In contrast, when BM–derived macrophages from WT and *Mlkl*^–/–^ mice were challenged with LPS, *Tnfa, Il1b,* and *Mcp1* mRNA expression was lower in cells from *Mlkl-*deficient mice compared with WT (Figure 5D). Taken together, these data suggest that *Mlkl*-independent expression of chemokines by hepatocytes, as well as neutrophil accumulation, sustains Gao-binge–induced injury, despite a lowering of some inflammatory cytokines.

### Plasma concentrations of RIP1, RIP3, and MLKL in patients with AH compared with NASH and HCs.

**Figure 6:**
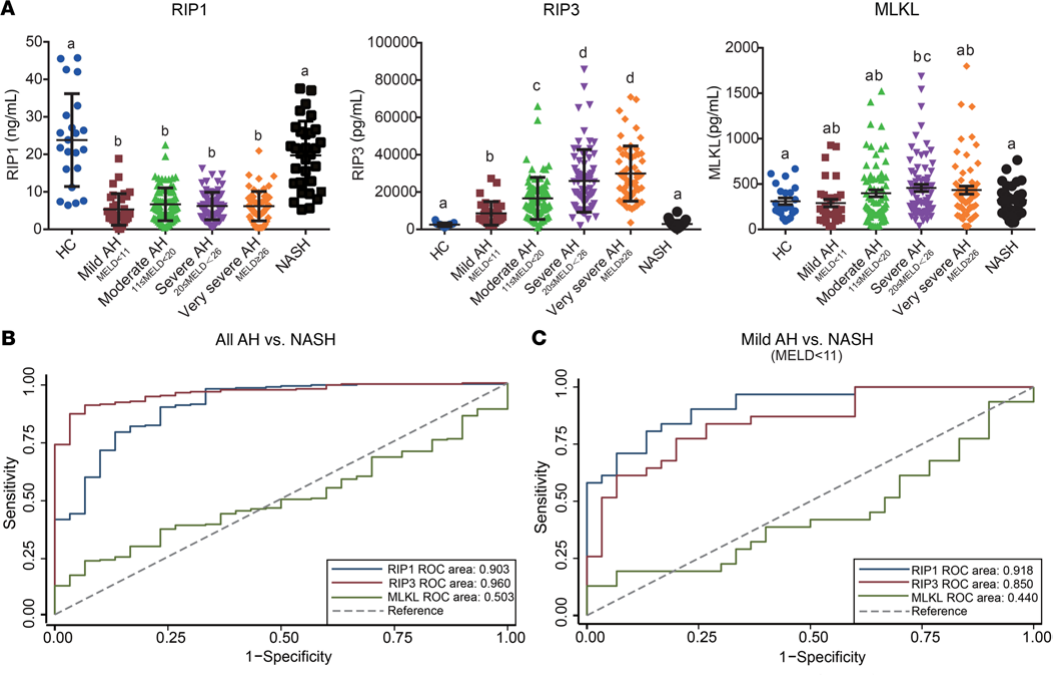
Quantity of RIP1, RIP3, and MLKL in plasma from HCs and patients with AH and NASH. (**A**) ELISA assays for RIP1, RIP3, and MLKL were conducted in plasma from HCs (*n* = 22) and patients diagnosed with NASH (*n* = 31), mild AH (MELD < 11, *n* = 25), moderate AH (11 ≤ MELD < 20, *n* = 83), severe AH (20 ≤ MELD < 26, *n* = 76), or very severe AH (MELD ≥ 26, *n* = 61). *P* < 0.05, assessed by 2-way ANOVA; values with different alphabetical superscripts were significantly different from each other. (**B**) Receiver operating characteristic (ROC) curves show the predictive values of RIP1, RIP3, and MLKL to distinguish all AH from NASH. (**C**) ROC curves show the predictive values of RIP1, RIP3, and MLKL to distinguish mild AH (MELD < 11) from NASH.

Previous reports suggest that circulating concentrations immunoreactive RIP1, RIP3, and MLKL, assessed by commercially validated ELISA kits, are associated with increased inflammation in lung injury (29) and sepsis (30). Although the cellular origin of circulating RIP1, RIP3, and MLKL is unknown, circulating biomarkers can still provide useful diagnostic indicators. Therefore, we measured RIP1, RIP3, and MLKL concentrations in plasma from HCs, patients with AH, stratified by disease severity based on the model for end-stage liver disease (MELD) score (mild AH [MELD < 11], moderate AH [11 ≤ MELD < 20], severe AH [20 ≤ MELD < 26], very severe AH [MELD ≥ 26]) and patients with NASH. The clinical characteristics of this cohort are shown in Table 2. Importantly, circulating concentrations of RIP1 and RIP3 were dependent on the etiology of liver disease in this cohort. RIP1 was lower in all AH patients independent of severity compared with both HCs and patients with NASH (Figure 6A). RIP3 was also higher in AH compared with HCs and NASH and increased with severity of AH (Figure 6A). In contrast, plasma MLKL concentration was not as responsive to disease severity or etiology (Figure 6A). RIP1 and RIP3, but not MLKL, were found to be good biomarkers for distinguishing AH from NASH (Figure 6B and Supplemental Table 3). To better compare the predictive value of RIP1 and RIP3 to distinguish AH from NASH, independent of disease severity, we also compared mild AH (MELD < 11) and NASH. Notably, subjects with similar MELD scores, RIP1, and RIP3 were still valuable in distinguishing AH from NASH (Figure 6C and Supplemental Table 3).

Recent data from Gautheron and colleagues found that the concentration of RIP1 and MLKL increased in patients with NASH dependent on disease severity (9), but this study only evaluated circulating RIP1 and MLKL in patients with different severities of NASH, without inclusion of HCs (9). They found that RIP 1 and MLKL concentrations were higher in patients with more severe NASH (activity score ≥ 2) compared with patients with milder NASH (activity score < 2) (9). We also analyzed RIP1, RIP3, and MLKL concentration according to NAFLD activity score (NAS) in patients with NASH (*n* = 30, 1 patient in our cohort did not have a recorded NAS), and found MLKL, but not RIP1 or RIP3, was higher in patients with NAS greater than or equal to 3 than those with NAS less than 3 (Supplemental Figure 2). Our cohort had only 2 patients with NAS less than 2, so, although there was a trend toward an increase, we could not statistically compare groups using NAS 2 as the cutoff value. Taken together, these data suggest that there are increases in circulating MLKL in patients with NASH as severity of disease increases, but the concentrations, even in more severe NASH, tend to be lower in patients with NASH compared with AH.

### Plasma concentrations of RIP1, RIP3, and MLKL and 90-day mortality in patients with AH.

**Figure 7:**
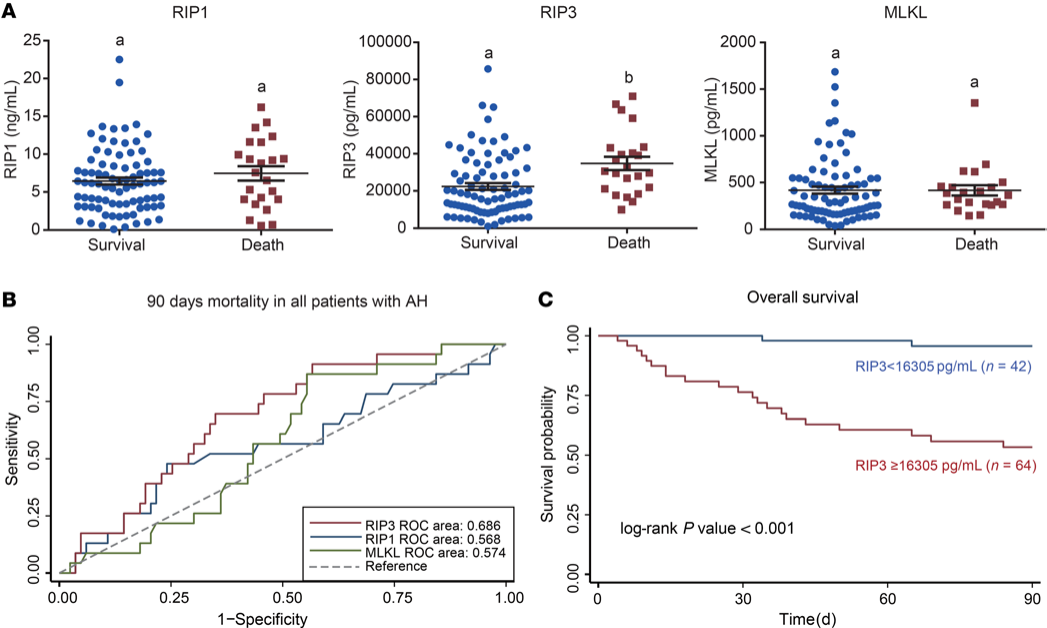
Association between the plasma concentrations of RIP1, RIP3, and MLKL and prognosis in patients with AH. (**A**) RIP3, not RIP1 and MLKL, can predict 90-day mortality in AH (survival *n* = 83, death *n* = 23). Values with different alphabetical superscripts were significantly different from each other. P less than 0.05, assessed by 2 tailed *t* test. (**B**) ROC curves show the predictive values of RIP1, RIP3, and MLKL to predict 90 days mortality in patients with AH. (**C**) The patients with high concentration of RIP3 (*n* = 64) have poor prognosis compared with those at a low concentration of RIP3 (*n* = 42) (log-rank P < 0.001). The best cutoff value was based on the calculation of Youden index, which takes into account the sensitivity and the specificity.

Since RIP1 and RIP3 were useful biomarkers to distinguish AH from NASH, we evaluated their ability to predict 90-day mortality in patients with AH who had survival data (*n* = 106). RIP3, but not RIP1 or MLKL, was different between patients who survived compared with those who died before 90 days (Figure 7A). In addition, RIP3 concentrations could better predict 90 days mortality in AH compared with RIP1 and MLKL (Figure 7B and Supplemental Table 3). By using the value 16,305 pg/mL as a cutoff value in RIP3, we found that patients with RIP3 concentrations above this cutoff had poorer prognoses after their diagnosis (Figure 7C), suggesting RIP3 may be a promising biomarker to predict prognosis in AH after diagnosis.

## Discussion

Multiple pathways of regulated cell death are implicated in the progression of metabolic liver diseases; however, the contributions of individual pathways of regulated cell death to specific liver diseases are not well understood (31). Studies using *Rip3*^–/–^ mice find that the contributions of RIP3 to liver injury vary considerably depending on the insult (4, 5, 7). Although RIP3 contributes to ethanol-induced liver injury (8, 11), as well as MCD-diet fed mice (14, 17), several studies using high-fat diet–induced models of NAFL/NASH find that *Rip3* does not contribute to liver injury (16, 18, 32). Although the role of RIP3 has been studied in multiple models of liver injury (4, 5, 7), much less is known about the role of MLKL, the downstream effector of necroptotic cell death, in liver disease. Recent studies revealed that *Mlkl*^–/–^ mice are protected from liver injury induced by an FFC diet (21), as well as high-fat diet–induced insulin resistance (20). Although no gold standard exists for assessing necroptosis, the clear involvement of *Mlkl* in murine models of NAFL/NASH implicates necroptotic cell death in disease development. If the contributions of RIP3 to ethanol-induced liver injury were due to activation of necroptosis, we would expect that *Mlkl*^–/–^ mice would be protected from injury. However, here we find that, in contrast to high-fat diet-induced obesity, *Mlkl* deficiency does not protect mice from ethanol-induced liver injury. Even in *Mlkl*-deficient mice, acute chronic ethanol exposure induced ER stress and hepatocellular injury, associated with increased *Cxcl1* and *Cxcl2* chemokine expression and neutrophil infiltration.

Additionally, we provide evidence that the concentration of circulating components of the necroptotic signaling network, RIP1 and RIP3, may provide important insight into the development of biomarkers to distinguish AH from NASH. Such biomarkers are becoming increasingly important as many patients presenting with symptoms of metabolic liver disease are both obese and chronic heavy drinkers (33). In our study, the circulating concentrations of RIP1 and RIP3 were different in patients with AH compared with NASH, even when AH patients were stratified by disease severity. Majdi and colleagues (9) reported that concentrations of RIP1 and MLKL were increased in serum of patients with NASH in patients with activity score greater than or equal to 2 compared with those with activity score less than 2. When we stratified our NASH cohort by disease severity (Supplemental Figure 2), our data were partially consistent with this report, in that the concentration of MLKL, but not RIP1 or RIP3, was higher in patients with NAS greater than or equal to 3 compared with those with NAS less than 3. Taken together, these data indicate that circulating MLKL is increased with NASH severity. Importantly, in our cohort of patients with AH and NASH, the RIP1 and RIP3 concentrations were still different between AH and NASH, even when stratified for disease severity based on MELD scores. Our data are consistent with previous work using circulating biomarkers of apoptosis in NAFL/NASH and AH. Feldstein and colleagues (34) originally developed CK18 as a biomarker for NAFL. CK18 is a cytokeratin exclusively expressed in hepatocytes within the liver. Release of full-length CK18 (M65) is indicative of necrotic/necroptotic cell death, whereas release of the caspase-dependent cleavage product M30 indicates caspase-dependent apoptosis of hepatocytes. In NAFL, the caspase-dependent cleavage product M30 predominates in the circulation (34), whereas in AH, M65 is predominant (35).

In murine models of metabolic liver disease, the expression, phosphorylation, and intracellular localization of MLKL was differentially regulated in response to Gao-binge ethanol compared with FFC diets. Although hepatic MLKL expression was relatively low in control mice, it was increased in response to FFC diets, but not Gao-binge. Increased expression in response to FFC diets, but not Gao-binge, was associated with increased phosphorylation of MLKL, oligomerization, and translocation to the PM. In FFC diets, accumulation of MLKL in liver is associated with impaired autophagic flux (21). Gao-binge ethanol also impairs autophagic flux, in a mechanism dependent on TFEB expression (36). However, our data suggest that impaired autophagic flux in the context of Gao-binge ethanol exposure is insufficient to result in the accumulation of MLKL in the liver. It is possible that impaired autophagic flux is more predominant in FFC diets and/or that there are additional stimuli involved in the induction of MLKL in response to high-fat diets.

When previous studies using global *Rip3*^–/–^ mice observed protection of liver injury in response to a variety of insults, including ethanol-induced liver injury (8, 11), acetaminophen-induced injury (12, 13), MCD diets (14), and concanavalin-A induced hepatitis (15), it was assumed that this protection was associated with a prevention of cell death receptor–mediated necroptotic cell death. However, RIP3 has a number of noncanonical functions that are independent of necroptosis, including activation of the inflammasome (37, 38). These functions are likely to be particularly important in the context of ethanol-induced liver injury in which the inflammasome is known to contribute to injury (39). Because MLKL is the ultimate downstream effector of necroptotic cell death, only protection from injury in the absence of MLKL can be a true indicator for a pathophysiological role of necroptosis in disease progression. Importantly, here we find that *Mlkl*^–/–^ are not completely protected from Gao-binge– or chronic ethanol-induced liver injury, suggesting that necroptosis is not an important driver of hepatocellular injury in this model of ethanol-induced liver injury.

However, it is notable that *Mlkl*-deficient mice were protected from some of the Gao-binge– induced increases in expression of some inflammatory mediators, including *Tnfa*, *Il1b*, and *Mcp1*, but not other chemokines, such as *Cxcl1* and *Cxcl2*. In the liver, both hepatocytes and immune cells contribute to increased inflammatory responses (40, 41). Hepatocytes are not typically considered to be important contributors to inflammation in the liver. However, hepatocytes respond to stress by activating multiple drivers of inflammation (42–44). For example, hepatocytes produce MCP1 and macrophage migration inhibitory factor, 2 important chemokines that contribute to ethanol-induced liver injury in murine models, in response to ethanol (45–47). Interestingly, using cultured monocytic cells, Kearney et al. reported that MLKL-dependent necroptotic cell death could limit inflammatory mediator production (48, 49). Further, Kang et al. (50) showed the IL1B production was suppressed by knockdown of MLKL in LPS-treated DCs. In contrast, Yoon and colleagues (51) found that *Mlkl* deficiency in HT29 colon cells did not impair TNF-stimulated chemokine production. Here, we also report that LPS-stimulated expression of *Cxcl1* and *Cxcl2,* important chemokines stimulating neutrophil recruitment (27), was also independent of *Mlkl* in AML12 hepatocytes. The sustained expression of *Cxcl1/2* was consistent with the *Mlkl*-independent accumulation of neutrophils in livers of Gao-binge ethanol–exposed mice. Taken together, these data suggest that there are likely cell-specific mechanisms of MLKL action in the liver in response to Gao-binge ethanol. This hypothesis will require studies using cell-specific deletions of *Mlkl* to distinguish the role of MLKL in hepatocytes versus immune cells.

Understanding the complex contributions of RIP1-RIP3 and MLKL in human disease is important, as necroptotic cell death has been associated with more than 30 human diseases (52), including cancer (53) and more than 20 approved drugs have the potential to regulate necroptosis (52). Taken together, our data in murine models and patients with AH and NASH suggest that circulating markers of specific cell death pathways would be potential biomarkers of the etiology of metabolic liver diseases. These data indicate that, given the difficulty of collecting biopsy samples in NASH and AH patients, development of RIP1, RIP3, and MLKL as a panel of less-invasive circulating biomarkers for NASH and AH may address an important unmet clinical need (54).

## Methods

### Human liver and plasma samples.

For Western blots and immunohistochemistry, samples from 5 livers explanted from patients with severe AH during liver transplantation and 5 wedge biopsies from healthy donor livers were snap frozen in liquid nitrogen and stored at –80°C. AH and healthy donor samples were provided by the NIAAA R24 Clinical Resource for Alcoholic Hepatitis Investigations at Johns Hopkins University. Samples from patients with NASH were obtained from 2 sources: wedge biopsies collected from 4 patients with NASH were provided by S. Dasarathy (NCT00323414) for Western blot analysis, and the liver sections used for immunohistochemistry from 16 patients with NAFL/NASH were obtained from the Cleveland Clinic Surgical Pathology CoPath database with the assistance of the Northern Ohio Alcohol Center. Descriptive demographic and clinical data from the patients used for Western blots are provided in Table 1.

For RIP1, RIP3, and MLKL ELISA assays, plasma from a total of 298 subjects was included in this study. Deidentified plasma samples, along with clinical and demographic data, were obtained from 22 healthy individuals and 31 patients with NASH from the Northern Ohio Alcohol Center biorepository (NCT03224949). Forty-four patients with AH were recruited from the Liver Unit of the Hospital Clínic Barcelona, between January 2000 and September 2007, and 201 patients with AH from 4 medical centers participating in the Defeat Alcoholic Steatohepatitis (DASH) consortium (Cleveland Clinic, University of Louisville School of Medicine, University of Massachusetts Medical School, and University of Texas Southwestern Medical Center) (55). Of the AH cohort from DASH consortium, 107 AH patients were followed for 180 days. Detailed descriptions of patient recruitment, inclusion and exclusion criteria for the Barcelona AH cohort (28), and DASH AH cohort (55) have been reported in previous studies. Patients with AH were stratified as mild AH (MELD < 11, *n* = 25), moderate AH (11 ≤ MELD < 20, *n* = 83), severe AH (20 ≤ MELD < 26, *n* = 76), and very severe AH (MELD ≥ 26, *n* = 61). Descriptive demographic and clinical data are provided in Table 2. This study was approved by the IRBs of all participating institutions, and all study participants consented prior to collection of data and blood samples.

### Animals and feeding trials.

All animals received humane care. *Mlkl^−/−^* mice were purchased from Taconic Biosciences (TF2780) and were backcrossed with C57BL/6J from The Jackson Laboratory (Bar Harbor, ME, USA) until congenic. C57BL/6J background was confirmed in the *Mlkl^−/−^* and *Mlkl^+/+^* using the SNP Genome Scan service at The Jackson Laboratory. For the ethanol feeding models, 8- to 10-week-old male and female mice were allowed free access to the Lieber-DeCarli high-fat ethanol diet or pair-fed control diets. Two ethanol feeding models, a chronic feeding model and the Gao-binge model, were used in this study. For the high-fat diet feeding studies, 5-week-old male mice were allowed free access to a diet high in FFC for 12 weeks as a model of high-fat diet–induced obesity; controls were fed standard chow diets. Additional details of the feeding protocols and tissue collection can be found in Supplemental Materials and Methods.

### Subcellular fractionation and PM isolation in murine liver.

PM fractions were isolated using the PM protein extraction kit (ab65400, Abcam). Liver tissues were washed twice with ice-cold PBS, resuspended in homogenization buffer and lysed using a Dounce homogenizer (50 strokes). Homogenates were centrifuged to obtain the cytosolic fraction as well as the 10,000*g* pellet. The pellet was further purified according to the manufacturer’s instructions to obtain purified PM proteins.

### Biochemical assays, histopathology, immunohistochemistry, Western blot, and ELISAs.

Detailed methods can be found in the Supplemental Materials and Methods; the lists of the primers for quantitative PCR (qPCR) and antibodies for WB are also available in the Supplemental Table 4 and 5.

### Statistics.

Values shown in all figures represent the means ± SEM. Values with different alphabetical superscripts were significantly different from each other. Statistical analysis was performed using SAS statistical software (SAS Institute) and STATA, version 16.0 (IBM). Data were log-transformed as necessary to obtain a normal distribution. Group comparisons were made by unpaired 2-tailed *t* test or 2-way ANOVA in continuous variables and by χ^2^ test or Fisher’s exact test where appropriate in categorical variables. ANOVA was performed using the general linear models procedure and follow-up comparisons made by least-squares means testing. The diagnostic accuracy for the diagnosis of AH, NASH, and prognostic accuracy of 90-day mortality of AH patients was evaluated using receiver operating characteristic curves. The leave-one-out cross-validation method was used to assess predictive potential and to limit model overfitting. The best cutoff values were based on the calculation of Youden index, which takes into account the sensitivity and the specificity. Kaplan-Meier survival curves were plotted to estimate the cumulative probability of mortality for RIP3 related to 90-day mortality in patients with AH from DASH consortium. A *P* value of less than 0.05 was considered significant.

### Study approval.

For human samples, written informed consent was obtained from all patients and all samples were deidentified. These studies were approved by the Institutional Review Boards at Johns Hopkins Medical Institutions, the Cleveland Clinic, MetroHealth Hospitals, or the Ethics Committee of the Hospital Clinic of Barcelona. Studies included in the DASH consortium were approved by the Institutional Review Boards at University of Massachusetts, University of Louisville, Cleveland Clinic, and University of Texas Southwest. All procedures using animals were approved by the Cleveland Clinic IACUC.

## Author contributions

TM, XW, and LEN contributed to the conception and design of the manuscript. TM, XW, XF, EH, CSG, CKCDR, AB, MRM, DSA, JC, PSB, CJM, MM, AJM, SR, BB, GS, and SD contributed to the collection and assembly of the data. JD and SD provided the liver samples. TM, XW, XF, EH, and SR contributed to the interpretation of the data and data analysis. TM, XW, MRM, and LEN contributed to the manuscript writing. All authors gave final approval of the manuscript.

## Supplementary Material

Supplemental data

## Figures and Tables

**Table 1:**
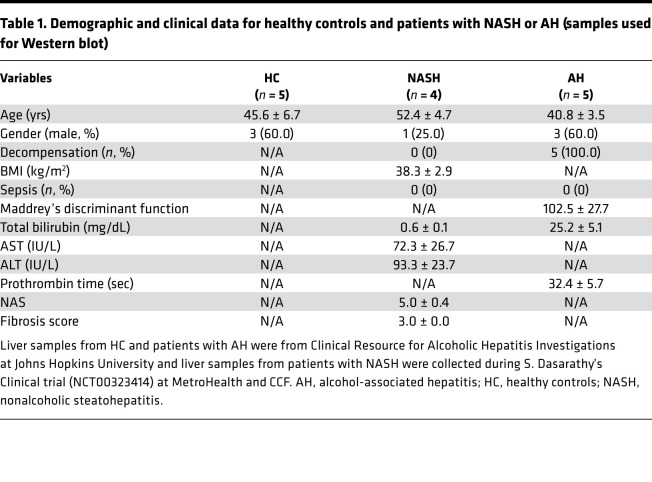
Demographic and clinical data for healthy controls and patients with NASH or AH (samples used for Western blot)

**Table 2:**
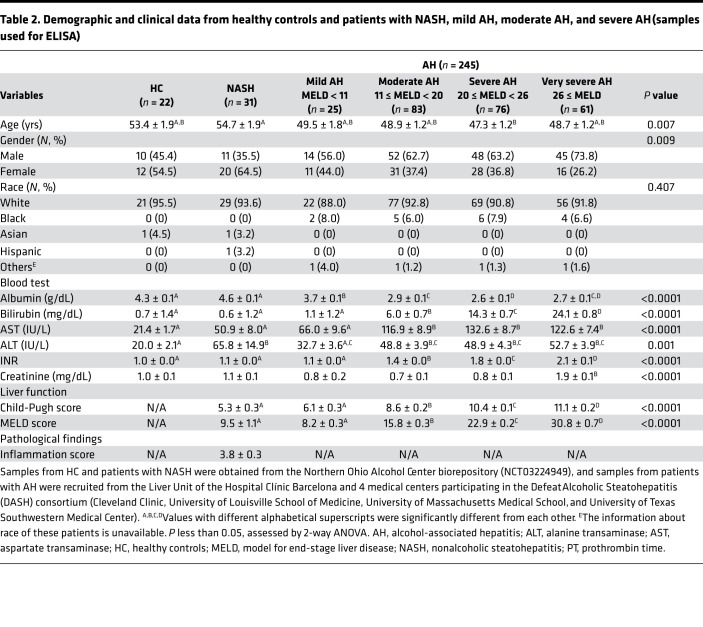
Demographic and clinical data from healthy controls and patients with NASH, mild AH, moderate AH, and severe AH (samples used for ELISA)

## Version 1 02/22/2021

Electronic publication

## Version 2 02/23/2021

Updated figures and PDF
