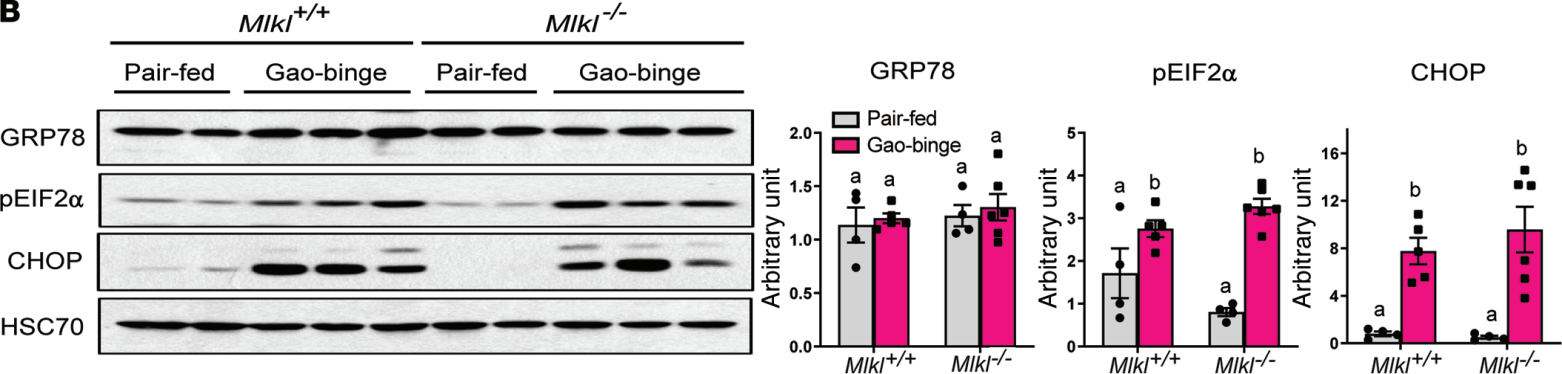# Differential role of MLKL in alcohol-associated and non–alcohol-associated fatty liver diseases in mice and humans

**DOI:** 10.1172/jci.insight.167011

**Published:** 2022-12-08

**Authors:** Tatsunori Miyata, Xiaoqin Wu, Xiude Fan, Emily Huang, Carlos Sanz-Garcia, Christina K. Cajigas-Du Ross, Sanjoy Roychowdhury, Annette Bellar, Megan R. McMullen, Jaividhya Dasarathy, Daniela S. Allende, Joan Caballeria, Pau Sancho-Bru, Craig J. McClain, Mack Mitchell, Arthur J. McCullough, Svetlana Radaeva, Bruce Barton, Gyongyi Szabo, Srinivasan Dasarathy, Laura E. Nagy

Original citation: *JCI Insight*. 2021;6(4):e140180. https://doi.org/10.1172/jci.insight.140180

Citation for this corrigendum: *JCI Insight*. 2022;7(23):e167011. https://doi.org/10.1172/jci.insight.167011

Following the publication of this article, the authors became aware of similarities between the CYP2E1 blot in Figure 4A and the pEIF2A blot in Figure 4B. After reviewing the original data, the authors determined that the pEIF2A blot in Figure 4B was incorrect and that this error was caused by misidentification of pEIF2A after reprobing of the CYP2E1 membrane for pEIF2A, CHOP, and GRP78. The authors have provided a correct version of Figure 4B with data obtained from a repeated experiment. This correct version appears below.

The authors regret the error.

## Figures and Tables

**Figure 4B:**