# Supplementary material for: Differential role of MLKL in alcohol-associated and non–alcohol-associated fatty liver diseases in mice and humans
Source: JCI Insight. 2021 Feb 22;6(4):e140180. doi: 10.1172/jci.insight.140180 (PMC7934930; doi:10.1172/jci.insight.140180)
Supplement: Supplemental data [file jciinsight-6-140180-s265.pdf]

Differential roles of MLKL in alcohol-associated liver disease and non-alcoholic fatty liver disease both in mice and human

Tatsunori Miyata*, Xiaoqin Wu*, Xiude Fan, Emily Huang, Carlos Sanz-Garcia, Christina K. Cajigas-Du Ross, Sanjoy Roychowdhury, Annette Bellar, Megan R. McMullen, Jaividhya Dasarathy, Daniela S. Allende, Joan Caballeria, Pau Sancho-Bru, Craig J. McClain, Mack Mitchell, Arthur J. McCullough, Svetlana Radaeva, Bruce Barton, Gyongyi Szabo, Srinivasan Dasarathy and Laura E. Nagy

Table of contents;

1. Supplemental Materials and Methods ------------------------------------- 1
2. Supplemental Figure 1 ------------------------------------- 5
3. Supplemental Figure 2 ------------------------------------- 6
4. Supplemental Table 1 ------------------------------------- 7
5. Supplemental Table 2 ------------------------------------- 8
6. Supplemental Table 3 ------------------------------------- 9
7. Supplemental Table 4 ------------------------------------- 10
8. Supplemental Table 5 ------------------------------------- 11

Supplemental Materials and Methods

Mouse feeding protocols

Chronic ethanol feeding model: 8-10 week old female mice were allowed free access to a Lieber-DeCarli liquid diet (Dyets, Bethlehem, PA, USA; Cat#710260) with increasing concentrations of ethanol: 1% and 2% (vol/vol) each for 2 days, then 4% and 5% ethanol each for 7 days, and finally 6% ethanol for an additional week [1, 2] . The 6% (vol/vol) diet provided ethanol as 32% of total calories in the diet. Control mice were pair-fed diets which iso-calorically substituted maltose dextrins for ethanol over the entire feeding period.

Gao-binge model (acute on chronic ethanol): 8-10 week old male and female mice were allowed free access to a Lieber-DeCarli liquid diet containing ethanol at 5% (v/v) or 28% of total calories or pair-fed a control diet that isocalorically substituted maltose dextrins for ethanol for 10 days. On the final day of the experiment, pair-fed mice were gavaged with 5g/kg maltose and ethanol-fed mice were gavaged with 5g/kg ethanol in water [3, 4] . Mice were euthanized 6 h after gavage.

FFC diet-induced obesity model: 5 week old male mice were allowed free access to a chow diet (Chow) containing 6% fat/13.0 kJ/g (#2918, Teklad Mills, Madison, WI) or the AIN-76A Western diet (#5342, TestDiet, St. Louis, MO, USA) for 12 weeks. The Western diet (FFC diet) contained 40% energy as fat (12% saturated fatty acid, 0.2% cholesterol) with fructose and glucose added to the water (42 g/L final concentration). At the end of the 12 weeks feeding protocol, mice were fasted for 4-6 hours prior to euthanasia [5, 6].

Mouse handling and tissue collection

Animals were housed in 2 per cage for ethanol feeding or 3 per cage for FFC feeding in standard microisolator cages and maintained in a temperature regulated facility with a 12h:12h light/dark cycle. In ethanol models, mice were weighed twice a week and food intake per cage measured daily, with Nylabones

provided for environmental enrichment. In high-fat models, mice were weighed and food intake per cage measured weekly, and water was changed twice a week.

At the end of the feeding protocols, mice were anesthetized, livers perfused with saline and then portions of liver were flash frozen in liquid nitrogen and stored at -80°C, fixed in 10% formalin or frozen in optimal cutting temperature (OCT) compound (Sakura Finetek U.S.A., Inc., Torrance, CA, USA) for histology. Blood was transferred to EDTA-containing tubes for the isolation of plasma. Plasma was then stored at -80°C.

Hepatocytes and hepatic non-parenchymal cells (NPC) were prepared by digesting livers in RPMI 1640/10% FBS with Type IV Collagenase (Sigma Aldrich, St. Louis, MO, USA, Lot# 087K8630) and DNase I (Roche, Mannheim, Germany) for 45min at 37° C. Digests were pressed through a 70 µm strainer and washed with RPMI 1640/10% FBS. Cells were centrifuged at 50 g for 2 min. Hepatocytes were collected from the pellet and further washed and then prepared for western blots. Supernatants from the 50 g spin were then centrifuged at 300 g for 15 min to collect NPC. Cells were resuspended in BD Pharm Lyse (BD Biosciences, San Jose, CA, USA) for 5 min on ice. Cells were washed with RPMI 1640/10% FBS and centrifuged at 300 g for 7 min. To enrich the NPC fraction, cells were centrifuged at 50 g for 10 min without brake in a 30% Percoll gradient. The cell layer was removed carefully and cells collected by centrifugation at 300 g for 10 min. Cells were washed with PBS and centrifuged at 300 g for 10 min and prepared for western blots.

AML-12 hepatocyte Cell Culture

The murine hepatocyte cell line, alpha mouse liver 12 (AML-12), was purchased through the American Tissue Culture Collection (ATCC) and grown in DMEM:F12 Medium supplemented with 10% fetal bovine serum, 10 µg/ml insulin, 5.5 µg/ml transferrin, 5 ng/ml selenium and 40 ng/ml dexamethasone (Complete medium). For experiments, AML12 hepatocyte were transfected with scrambled siRNA or siRNA targeted to knock-down *Mlkl*. AML12 hepatocyte

were acclimated to medium with 2.5% FBS for 18 h prior to stimulation with bacterial lipopolysaccharide (LPS) (Thermo Fisher Scientific, Waltham, MA, USA). AML12 hepatocytes were treated with LPS (10ng/mL) for 90 mins, and their total RNA isolated.

Preparation of bone marrow-derived macrophages (BMDMs)

Primary cultures of BMDMs were generated from tibia and fibula of WT and *Mlkl*$^{-/-}$ mice. BM cells were cultured with DMEM supplemented with 10% FBS and 20 ng/mL macrophage colony stimulating factor (M-CSF) at 37°C/5% CO_2. Fresh culture medium was replaced on day 3, 6 and 9. On day 10, adherent cells were seeded and then treated with LPS (10 ng/mL). 24 h later, RNA was isolated to measure cytokine/chemokine expression.

Biochemical assays

Plasma samples were assayed for alanine aminotransferase (ALT) and aspartate aminotransferase (AST) using commercially available enzymatic assay kits (Sekisui Diagnostics, Seacacus, NJ, USA) following the manufacturer's instructions. Total hepatic triglycerides were assayed using the Triglyceride Reagent Kit from Pointe Scientific Inc. (Lincoln Park, MI, USA).

Histopathology and immunohistochemistry

Formalin-fixed liver tissues were paraffin-embedded, sectioned and stained with hematoxylin and eosin for histological analysis. Formalin-fixed paraffin-embedded mouse liver sections were de-paraffinized and used to assess the caspase-generated fragment of cytokeratin-18 (M-30) (12140322001, Roche, Indianapolis, IN, USA), NIMP-R14 (ab2557) and pMLKL (S345) (ab196436, Abcam, Cambridge, MA, USA) staining. Formalin-fixed paraffin-embedded human liver sections were de-paraffinized and used to assess pMLKL (Thr357, 954724) (mab91871, Novus Biologicals, Centennial, CO, USA). Nuclei were counter-stained with hematoxylin. Tissues were coded at time of collection to assure an un-biased analysis; at least 3 images were acquired per tissue section and semi-quantification of positive staining was performed using ImagePro Plus software (Media Cybernetics, Silver Springs, MD, USA). No

specific immunostaining for M30 and pMLKL was detected in liver sections incubated with PBS rather than the primary antibody (data not shown).

Isolation of RNA and quantitative real-time polymerase chain reaction (qRT-PCR)

Total RNA was isolated from liver tissue, AML12 and BMDM, and reverse transcribed followed by amplification using qRT-PCR. 10μl of reaction mix contained cDNA, Power SYBR Buffer (Applied Biosciences) and primers at final concentrations of 1-μM. qRT-PCR was performed in a Quantstudio 5 System for 40 cycles of 15 s at 95°C, 30 s at 60°C, 30 s at 72°C. The relative amount of target mRNA was determined using the comparative threshold (Ct) method by normalizing target mRNA Ct values to those of *18s*. Primer sequences are listed in **Supplemental Table 4**.

Western blot analysis

Frozen liver tissues (50-80mg) from mice were homogenized in lysis buffer (10 ml/ g tissue) and protein concentration measured using the DC Lowry assay [7]. Samples were prepared in Laemmli buffer and denatured at 37° for 15 min for RIP1, RIP3 and MLKL; samples for all other proteins were denatured by boiling for 5 min. Proteins were separated on 8 to 10% gels by SDS-PAGE and liver lysates were used for Western blot analysis against antibodies described in **Supplemental Table 5**. Western blot analysis was performed using enhanced chemiluminescence for signal detection. Signal intensities were quantified by densitometry using Image J software (NIH, Bethesda, MD, USA).

Measurement of RIP1, RIP3 and MLKL in human plasma

Human plasma samples were collected and stored at -80℃. Plasma RIP1, RIP3 and MLKL concentrations were quantified by ELISA using the following kits: human RIP1 ELISA Kit (LS-F9254, LSBio, Seattle, WA, USA), human RIP3 ELISA Kit (CSB-EL019737HU, CUSABIO, Houston, TX, USA) and human MLKL ELISA Kit (MBS9300811, MyBiosource, San Diego, CA, USA) and according to the manufacturer’s instructions.

Supplemental Figures

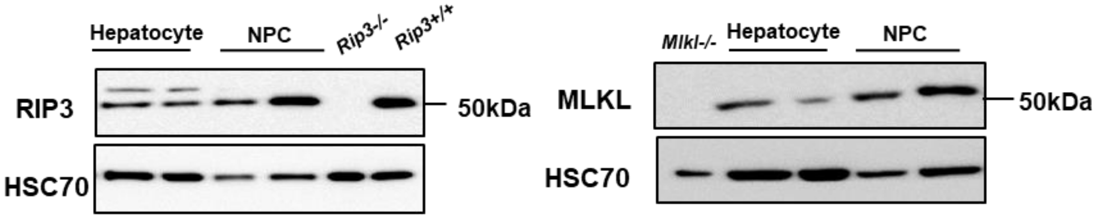

Figure S1. Basal expression of RIP3 and MLKL in isolated hepatocytes and non-parenchymal cells (NPC). Western blot analysis of RIP3 and MLKL expression in isolated hepatocytes and NPC from wild-type C57BL6J mice. Liver lysates from *Mlkl* and *Rip3*-deficient mice were used as negative controls

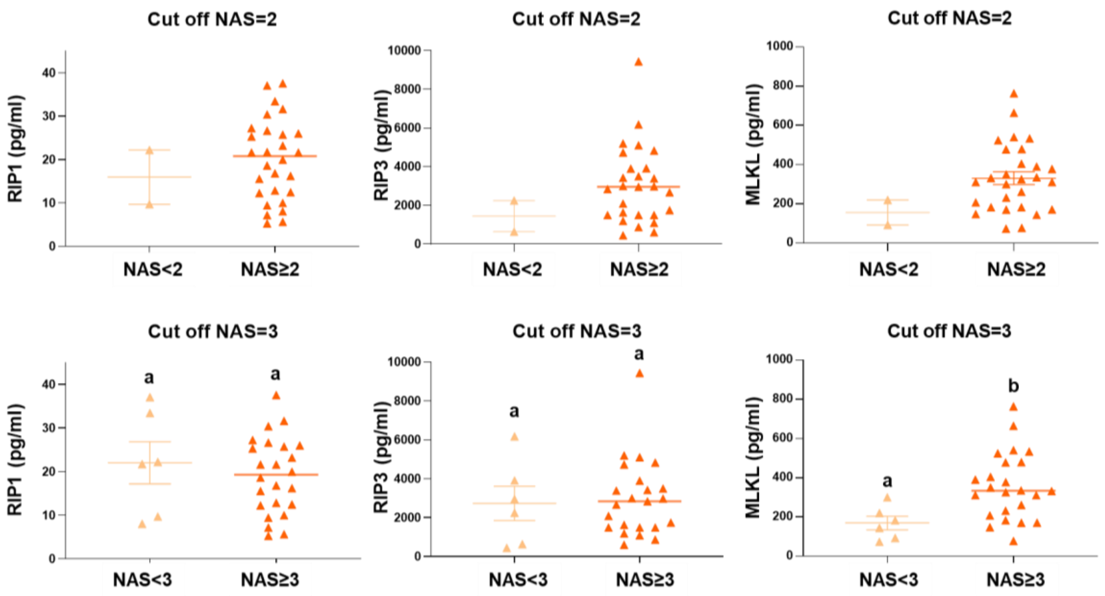

Figure S2. Concentration of RIP1, RIP3 and MLKL in human plasma in patients with NASH. ELISA analysis of RIP1, RIP3 and MLKL in plasma in patients with NASH (n=30). Using a cut-off of NAS2, as was used in Majdi, et al., we only had 2 patients in our cohort, so did not conduct a statistical analysis. Using a cut-off of NAS3, RIP1, RIP3 and MLKL were compared by t-test (p=0.014 for MLKL).

Supplemental Table 1. Body weights and food intakes for mice on the Gao-binge and chronic ethanol diets.

**Gao-binge Model (female)***					
Genotype	Pair-fed		Gao-binge		Gao-binge
	**Initial body weight (g)**	**Final body weight (g)**	**Initial body weight (g)**	**Final body weight (g)**	**Average daily food intake (mL/cage)**
Mlkl$^{+/+}$	20.2 ± 0.3	21.6 ± 0.5	20.6 ± 0.2	20.5 ± 0.5	20.3 ± 1.7
Mlkl$^{-/-}$	18.9 ± 0.4	20.2 ± 0.4	19.0 ± 0.2	19.7 ± 0.5	20.6 ± 1.5

**Gao-binge Model (male)†**					
Genotype	Pair-fed		Gao-binge		Gao-binge
	**Initial body weight (g)**	**Final body weight (g)**	**Initial body weight (g)**	**Final body weight (g)**	**Average daily food intake (mL/cage)**
Mlkl$^{+/+}$	25.5 ± 1.0	29.0 ± 1.3	25.6 ± 0.4	26.5 ± 0.7	21.4 ± 0.9
Mlkl$^{-/-}$	25.8 ± 0.6	27.1 ± 0.7	25.8 ± 0.5	24.7 ± 0.7	22.2 ± 1.7

**Chronic Ethanol Model (female)†**					
Genotype	Pair-fed		EtOH-fed		EtOH-fed
	**Initial body weight (g)**	**Final body weight (g)**	**Initial body weight (g)**	**Final body weight (g)**	**Average daily food intake (mL/cage)**
Mlkl$^{+/+}$	19.3 ± 1.2	22.0 ± 1.3	19.4 ± 0.5	20.9 ± 0.5	20.6 ± 0.7
Mlkl$^{-/-}$	18.9 ± 0.6	21.2 ± 0.1	19.2 ± 0.1	21.3 ± 0.3	20.7 ± 0.9

Values represent means ±SEM

*Data are from two independent feeding trial, n = 8-12 animals per group.

†Data are from one feeding trial, n = 4-6 animals per group.

Supplemental Table 2. Histological scoring of livers from pair-fed and Gao-binge mice

Variables	*Mlkl*$^{+/+}$		*Mlkl*$^{-/-}$	
	Pair-fed	Gao-binge	Pair-fed	Gao-binge
Female				
Steatosis (%)	0 ± 0	20.0 ± 6.3^{a}	0 ± 0	8.3 ± 2.8^{a}
Inflammation score	0 ± 0	0.4 ± 0.2^{a}	0.5 ± 0.3	1.0 ± 0^{b}
Ballooning	0 ± 0	0 ± 0	0 ± 0	0 ± 0
NAS	0 ± 0	1.6 ± 0.4^{a}	0.5 ± 0.3	1.8 ± 0.2^{a}
Male				
Steatosis	5.0 ± 3.5	28.3 ± 2.8^{a}	0 ± 0	27.5 ± 10.5^{a}
Inflammation score	0.5 ± 0.3	0.3 ± 0.2^{a}	0 ± 0	0.2 ± 0.2^{a}
Ballooning	0 ± 0	0 ± 0^{a}	0 ± 0	0.2 ± 0.2^{a}
NAS	1.0 ± 0.4	1.7 ± 0.3^{a}	0.3 ± 0.3	1.7 ± 0.7^{a}

Abbreviations; NAS: NAFLD activity score. Values represent means ± SEM. Values from Gao-binge groups with different alphabetical superscripts were significantly different from each other. n=4-6. P<0.05 asessed by t-test.

Supplemental Table 3 Diagnostic capacity of RIP1/RIP3/MLKL

For the identification of all patients with AH from patients with NASH

**Variable**	**AUC**	**95%CI**	**P value**	**Cut-off**	**Sensitivity(%)**	**Specificity(%)**
RIP1 (ng/ml)	0.903	0.848-0.965	0.030	9.4	90	77
RIP3 (pg/ml)	0.960	0.935-0.984	0.013	6181.8	90	93
MLKL (pg/ml)	0.503	0.416-0.590	0.044	540.8	28	93

For the identification of patients with mild AH (MELD<11) from patients with NASH

**Variable**	**AUC**	**95%CI**	**P value**	**Cut-off**	**Sensitivity (%)**	**Specificity (%)**
RIP1 (ng/ml)	0.918	0.864-0.985	0.031	9.3	81	85
RIP3 (pg/ml)	0.850	0.754-0.945	0.049	3965.5	81	80
MLKL (pg/ml)	0.440	0.277-0.564	0.073	778.5	45	33

For the 90 days mortality

**Variable**	**AUC**	**95%CI**	**P value**	**Cut-off**	**Sensitivity (%)**	**Specificity (%)**
RIP1 (ng/ml)	0.568	0.425-0.710	0.073	8.7	48	76
RIP3 (pg/ml)	0.686	0.573-0.798	0.057	16305.4	91	48
MLKL (pg/ml)	0.574	0.458-0.690	0.059	257.3	87	45

Supplemental Table 4. Primer Pairs for Real Time qPCR Analysis of Gene Expression

**Gene**	**Forward Sequence**	**Reverse Sequence**
Tnfa	CCCTCACACTCAGATCATCTTCT	GCTACGACGTGGGCTACAG
Il1b	ATGGCAACTGTTCCTGAACTCAACT	CAGGACAGGTATAGATTCTTTCCTTT
Mcp1	AGGTCCCTGTCATGCTTCTG	TCTGGACCCATTCCTTCTTG
Cxcl1	TGCACCCAAACCGAAGTC	GTCAGAAGCCAGCGTTCACC
Cxcl2	GCGCCCAGACAGAAGTCATAG	AGCCTTGCCTTTGTTCAGTATC
18s	ACGGAAGGGCACCACCAGGA	CACCACCACCCACGGAATCG

Supplemental Table 5. Antibodies used for western blots

**Antibody**	**Catalog No.**	**Company**
For human		
phospho-MLKL (S358)	ab187091	Abcam, Cambridge, MA
MLKL	ab184718	Abcam, Cambridge, MA
RIP3	XA-1023	ProSci, Poway, CA
GAPDH	MAB374	EMD Millipore, Burlinton, MA
For mouse		
CYP2E1	AB1252	EMD Millipore, Burlinton, MA
phospho-EIF2A (Ser51)	3597	Cell Signaling, Danvers, MA
CHOP	5554	Cell Signaling, Danvers, MA
GRP78	610978	BD Transduction, San Jose, CA
MLKL	66675	Proteintech, Rosemont, IL
phospho-MLKL (S345)	ab196436	Abcam, Cambridge, MA
HSC70	sc-7298	Santa Cruz Biotechnology, Dallas, TX
GAPDH	MAB374	EMD Millipore, Burlinton, MA

Reference List

1. Roychowdhury S, McMullen MR, Pisano SG, Liu X, Nagy LE. Absence of receptor interacting protein kinase 3 prevents ethanol-induced liver injury. *Hepatology (Baltimore, Md)*. May 2013;57(5):1773-83. doi:10.1002/hep.26200

2. Roychowdhury S, McMullen MR, Pritchard MT, Li W, Salomon RG, Nagy LE. Formation of gamma-ketoaldehyde-protein adducts during ethanol-induced liver injury in mice. *Free radical biology & medicine*. Dec 1 2009;47(11):1526-38. doi:10.1016/j.freeradbiomed.2009.07.015

3. Bertola A, Mathews S, Ki SH, Wang H, Gao B. Mouse model of chronic and binge ethanol feeding (the NIAAA model). *Nature protocols*. Mar 2013;8(3):627-37. doi:10.1038/nprot.2013.032

4. Sanz-Garcia C, et al. The non-transcriptional activity of IRF3 modulates hepatic immune cell populations in acute-on-chronic ethanol administration in mice. *Journal of hepatology*. May 2019;70(5):974-984. doi:10.1016/j.jhep.2019.01.021

5. Ibrahim SH, Hirsova P, Malhi H, Gores GJ. Animal Models of Nonalcoholic Steatohepatitis: Eat, Delete, and Inflame. *Digestive diseases and sciences*. May 2016;61(5):1325-36. doi:10.1007/s10620-015-3977-1

6. Wu X, et al. MLKL-dependent signaling regulates autophagic flux in a murine model of non-alcoholic fatty liver disease. *Journal of hepatology*. Mar 24 2020;doi:10.1016/j.jhep.2020.03.023

7. Zhou H, et al. IRAKM-Mincle axis links cell death to inflammation: Pathophysiological implications for chronic alcoholic liver disease. *Hepatology (Baltimore, Md)*. Dec 2016;64(6):1978-1993. doi:10.1002/hep.28811
